# Admission Glucose and Presenting Symptoms in Relation to Disease Stage in Newly Diagnosed Pancreatic Ductal Adenocarcinoma

**DOI:** 10.3390/jcm15155965

**Published:** 2026-07-30

**Authors:** Bartłomiej Węglarz, Leszek Czupryniak

**Affiliations:** Department of Diabetology and Internal Medicine, Medical University of Warsaw, 02-091 Warsaw, Poland; bartlomiej.weglarz@uckwum.pl

**Keywords:** pancreatic ductal adenocarcinoma, admission glucose, hyperglycaemia, jaundice, disease stage, metastasis, logistic regression, ROC analysis

## Abstract

**Background/Objectives:** Pancreatic ductal adenocarcinoma (PDAC) carries the poorest prognosis among gastrointestinal malignancies, largely owing to diagnosis at an advanced stage. Hyperglycaemia and new-onset diabetes are increasingly recognised as early manifestations of PDAC, yet long-standing type 2 diabetes is also an established risk factor. Whether admission plasma glucose, together with presenting symptoms, are associated with disease stage at diagnosis remain poorly characterised. We examined the association of admission glucose and presenting symptoms with disease stage in newly diagnosed PDAC. **Methods:** We performed a retrospective, single-centre analysis of 114 consecutive patients with histologically confirmed PDAC and both recorded admission plasma glucose and imaging-confirmed stage. Random admission plasma glucose, diabetes status, and presenting symptoms were extracted from electronic medical records. Symptoms were categorised as jaundice, abdominal pain, weight loss, or incidental (asymptomatic) detection. Disease stage was classified as metastatic (M1) or non-metastatic (M0) on the basis of imaging at diagnosis. Groups were compared using the Mann–Whitney U test and the χ^2^ test with continuity correction; multivariable logistic regression and receiver operating characteristic (ROC) analysis were performed. **Results:** The cohort comprised 60 women (52.6%) and 54 men (47.4%); 49 patients (43.0%) had metastatic and 65 (57.0%) had non-metastatic disease. Median age at diagnosis was 68 years (range 27–88). Diabetes was present in 40.4% (46/114), predominantly type 2 (87%), and did not differ by stage (42.9% vs. 38.5%; *p* = 0.78). Jaundice was more frequent in metastatic than non-metastatic disease (59.2% vs. 38.5%; χ^2^ = 4.02, *p* = 0.045), whereas abdominal pain (53.1% vs. 36.9%; *p* = 0.13) and weight loss (24.5% vs. 33.8%; *p* = 0.38) did not differ significantly. Mean admission glucose was 145.5 ± 59.8 mg/dL and was higher in patients with than without diabetes (178.6 ± 68.7 vs. 123.2 ± 40.1 mg/dL; *p* < 0.0001). Glucose analysed continuously did not differ by stage (148.4 vs. 143.3 mg/dL; *p* = 0.29), whereas hyperglycaemia ≥ 126 mg/dL was more frequent in metastatic disease (63.3% [95% CI 49.3–75.3] vs. 41.5% [95% CI 30.4–53.7]; χ^2^ = 4.44, *p* = 0.035; odds ratio 2.42, 95% CI 1.13–5.19). In multivariable logistic regression adjusting for age, sex, and diabetes, hyperglycaemia remained independently associated with metastatic stage (adjusted odds ratio 2.59, 95% CI 1.13–5.95; *p* = 0.025). However, discrimination was poor: the area under the ROC curve for admission glucose was 0.56 (95% CI 0.45–0.66). Among jaundiced patients, admission glucose did not differ significantly by stage (162.8 vs. 152.8 mg/dL; *p* = 0.41). **Conclusions:** In this retrospective, single-centre cohort, jaundice at presentation and admission hyperglycaemia ≥ 126 mg/dL were each associated with metastatic stage, and hyperglycaemia remained associated after adjustment. Nevertheless, the discriminative performance of admission glucose was poor, and these variables cannot be recommended for individual patient stratification. These hypothesis-generating findings require confirmation in larger, prospective, multicentre studies.

## 1. Introduction

Pancreatic cancer is among the most lethal malignancies, characterised by late detection and high mortality. In Poland, pancreatic cancer is a leading cause of cancer death, and national registry data place it among the most frequent causes of cancer mortality, underscoring its public-health burden [1].

This tumour is frequently diagnosed at an advanced stage, which substantially limits the prospect of effective treatment [2,3]. Early symptoms are typically non-specific and may be mistaken for other conditions; the literature describes abdominal pain, weight loss, jaundice, and dyspepsia as common presenting features [4].

Disturbances of glucose homeostasis are a well-recognised feature of PDAC, and their pathogenesis is complex and bidirectional. Long-standing type 2 diabetes is an established risk factor for PDAC, plausibly through chronic hyperinsulinaemia, insulin resistance, and a pro-inflammatory milieu that favours tumour growth [5]. Conversely, new-onset diabetes may represent an early paraneoplastic manifestation of an existing tumour rather than a cause: PDAC cells and the surrounding stroma release mediators, including adrenomedullin and the S100A8 N-terminal peptide, that impair β-cell function and induce peripheral insulin resistance [6,7]. Progressive destruction and fibrosis of pancreatic parenchyma further reduce insulin secretory reserve, producing a pancreatogenic (type 3c) pattern of diabetes [8]. Because these mechanisms may intensify as tumour burden increases, glucose concentrations measured at presentation could plausibly reflect the extent of disease [9,10].

Random admission plasma glucose is an attractive candidate marker in this context because it is measured routinely in virtually every hospitalised patient, requires no fasting, incurs negligible additional cost, and is available at the point of first contact—unlike fasting glucose, HbA1c, or specialised tumour markers, which are obtained inconsistently in the acute setting [9,11]. We therefore hypothesised that admission plasma glucose and the pattern of presenting symptoms differ between patients with metastatic and non-metastatic PDAC at diagnosis. The aim of this study was to characterise the presenting symptoms and admission glucose of patients with newly diagnosed PDAC treated at a clinical hospital in Warsaw from September 2024 to August 2025, and to assess whether these routinely available variables are associated with disease stage at diagnosis. Given the retrospective design, the analysis was exploratory and hypothesis-generating rather than intended to establish diagnostic or prognostic utility [12].

## 2. Materials and Methods

### 2.1. Study Design and Setting

This was a retrospective cohort study of patients treated across the clinical units of the University Clinical Centre of the Medical University of Warsaw (UCK WUM). The primary objective was to characterise the spectrum of presenting symptoms and admission glucose in patients with confirmed pancreatic cancer from September 2024 to August 2025. All consecutive patients meeting the eligibility criteria during the study period were included; no sampling or matching was applied. No a priori sample size calculation was performed, as the study was limited to the fixed number of eligible cases available at a single centre during the observation period; the resulting sample size is therefore a constraint of the available data rather than a powered design, and is addressed in Section 4.6. The analysis was based exclusively on electronic medical records held in the hospital information system (CGM Clininet, CompuGroup Medical Polska Sp. z o.o., Warsaw, Poland), the institution’s principal platform for clinical data. The study was reported in accordance with the Strengthening the Reporting of Observational Studies in Epidemiology (STROBE) statement.

### 2.2. Eligibility Criteria

Eligible patients were adults (≥18 years) with histopathologically confirmed pancreatic ductal adenocarcinoma who were hospitalised within UCK WUM during the study period. Patients were excluded if they met any of the following criteria: (i) a coexisting active malignancy of another primary site; (ii) a pancreatic tumour of non-ductal histology (for example, neuroendocrine tumour, acinar cell carcinoma, or intraductal papillary mucinous neoplasm without invasive adenocarcinoma); (iii) documented acute pancreatitis, sepsis, or diabetic ketoacidosis at the time of admission, each of which may substantially alter admission glycaemia; (iv) treatment with systemic glucocorticoids at admission; (v) PDAC diagnosed before the start of the observation period; or (vi) incomplete records, defined as absence of a recorded admission plasma glucose value or absence of imaging permitting classification of metastatic status. Of the 115 patients with histologically confirmed PDAC and imaging-confirmed stage during the study period, one was excluded because no admission plasma glucose value was recorded, yielding a final analytic cohort of 114 patients.

### 2.3. Data Extraction and Quality Control

Eligibility was assessed independently by two investigators, each evaluating every case against the predefined criteria; discrepancies were resolved by consensus after joint review. A standardised extraction form was applied consistently throughout data collection, with defined fields for all study variables and instructions for symptom classification and interpretation of laboratory results. Cross-verification of key clinical parameters and multi-step checking of entered values were used to minimise systematic error.

Extracted variables comprised age, sex, diabetes status and treatment, random admission plasma glucose, the initial presenting symptoms, and the results of ancillary investigations, in particular diagnostic imaging permitting classification of stage (M0 vs. M1). Where a biochemical parameter had been determined repeatedly during hospitalisation, only the first value obtained at admission was considered valid for analysis; the admission sample was defined as the first venous blood sample drawn within 24 h of hospital admission and before any therapeutic intervention, including intravenous fluids containing glucose. Plasma glucose was measured in the central hospital laboratory by the enzymatic hexokinase method on automated clinical chemistry analysers (Cobas c503, Roche Diagnostics GmbH, Mannheim, Germany; and Alinity c, Abbott Laboratories, Abbott Park, IL, USA), with a laboratory reference range of 70–99 mg/dL for fasting plasma glucose, hyperglycaemia was defined a priori as admission glucose ≥ 126 mg/dL. Jaundice was recorded when clinically documented icterus or scleral icterus was accompanied by biochemical cholestasis, defined as elevated total bilirubin with a predominantly conjugated fraction together with elevation of alkaline phosphatase and γ-glutamyl transferase above the upper reference limit; in this cohort jaundice was of obstructive (post-hepatic) origin in all cases, with imaging confirmation of biliary duct dilatation or tumour-related biliary obstruction, and no cases of purely intrahepatic cholestasis without ductal obstruction were identified. Presenting symptoms were categorised as jaundice, abdominal pain, weight loss, or incidental detection; categories were not mutually exclusive, and patients could present with more than one symptom. Incidental detection was defined as PDAC identified on imaging performed for an unrelated indication, or during evaluation for another condition, in a patient without jaundice, abdominal pain, or weight loss attributable to the tumour. Disease stage was classified as metastatic (M1) or non-metastatic (M0) on the basis of contrast-enhanced computed tomography of the chest, abdomen, and pelvis in all patients, supplemented where clinically indicated by magnetic resonance imaging, endoscopic ultrasound, or ^18^F-fluorodeoxyglucose positron emission tomography/computed tomography.

### 2.4. Statistical Analysis

Continuous variables were summarised as mean ± standard deviation and as median with range or interquartile range. Categorical variables were expressed as counts and percentages. The distribution of continuous variables was assessed using the Shapiro–Wilk test; admission plasma glucose was markedly right-skewed (*p* < 0.001), and continuous comparisons between the metastatic and non-metastatic groups were therefore performed using the Mann–Whitney U test. Categorical variables were compared using the χ^2^ test with a continuity (Yates) correction for 2 × 2 comparisons. Proportions are reported with 95% confidence intervals (CI) calculated by the Wilson score method, and associations as odds ratios (OR) with 95% CI. To assess whether hyperglycaemia was independently associated with metastatic stage, multivariable logistic regression was performed with metastatic disease (M1) as the dependent variable and hyperglycaemia ≥ 126 mg/dL, age, sex, and diabetes status as covariates; a secondary model additionally included jaundice, and a sensitivity model replaced categorical hyperglycaemia with continuous glucose (per 10 mg/dL). Results are presented as adjusted odds ratios (aOR) with 95% CI. The discriminative performance of admission glucose for metastatic stage was assessed by receiver operating characteristic (ROC) analysis; the area under the curve (AUC) is reported with a bootstrap 95% CI (2000 resamples), and the Youden index was used to identify the empirically optimal cut-off. A two-sided *p* < 0.05 was considered statistically significant. No adjustment for multiple comparisons was applied, and analyses should be interpreted as exploratory. Analyses were performed using TIBCO Statistica version 13.3 (TIBCO Software Inc., Palo Alto, CA, USA).

## 3. Results

### 3.1. Cohort Characteristics

Of 115 consecutive patients with histologically confirmed PDAC and imaging-confirmed stage identified during the study period, 114 had recorded admission plasma glucose and constituted the analytic cohort. The cohort comprised 60 women (52.6%) and 54 men (47.4%). Forty-nine patients (43.0%) had metastatic disease (M1) and 65 (57.0%) had non-metastatic disease (M0). Median age at diagnosis was 68 years (range 27–88). The two stage groups were comparable with respect to age (median 68 vs. 68 years; *p* = 0.98), male sex (46.9% vs. 47.7%; *p* = 1.00), and the prevalence of diabetes (42.9% vs. 38.5%; *p* = 0.78), indicating that these variables were unlikely to confound the stage comparisons. Baseline characteristics are summarised in Table 1.

### 3.2. Metabolic Characteristics

Diabetes was present in 46 patients (40.4% of the cohort), predominantly type 2 (40/46, 87%); three patients had type 1 diabetes and two had pancreatogenic (type 3c) diabetes. Among patients with diabetes, treatment consisted of oral glucose-lowering agents in 31, insulin with or without oral agents in nine, and diet alone in one, while treatment was not documented in five. Mean admission plasma glucose for the whole cohort was 145.5 ± 59.8 mg/dL (median 127 mg/dL, interquartile range 101–166). Glucose was significantly higher in patients with diabetes than without (178.6 ± 68.7 vs. 123.2 ± 40.1 mg/dL; *p* < 0.0001), consistent with a strong association between coexisting diabetes and admission hyperglycaemia [6]. The duration of diabetes was documented in only nine of 46 patients and HbA1c was available in only six of 114 patients overall; consequently, new-onset and long-standing diabetes could not be reliably distinguished, and no analysis stratified by diabetes duration or glycaemic control was undertaken. This limitation is considered further in Section 4.6.

### 3.3. Presenting Symptoms

**Figure 1:**
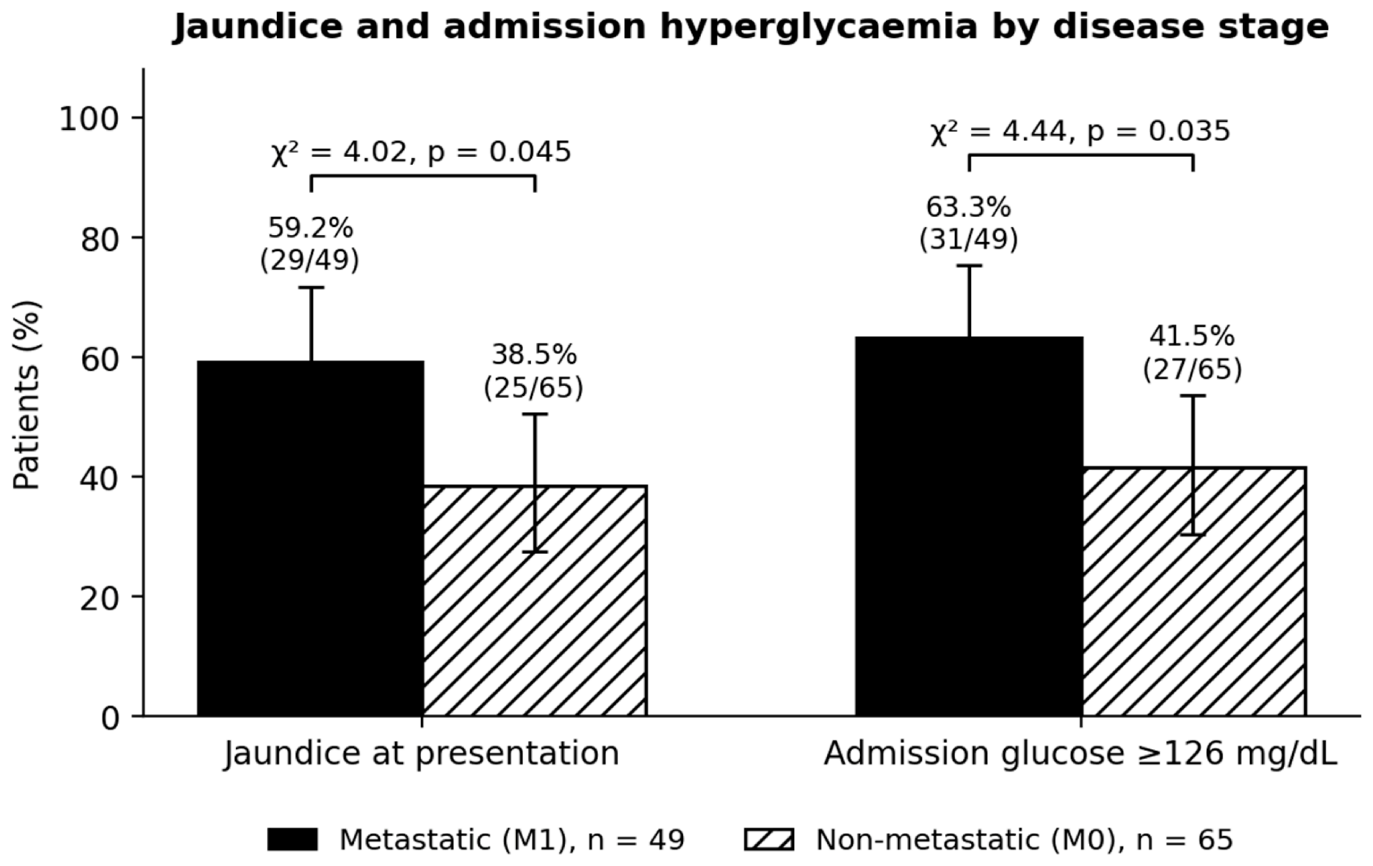
Frequency of jaundice at presentation and of admission hyperglycaemia (glucose ≥ 126 mg/dL) by disease stage. Bars show percentages within each stage group with 95% confidence intervals (Wilson score method); absolute counts are given above each bar. Jaundice: 59.2% vs. 38.5% (χ^2^ = 4.02, *p* = 0.045). Hyperglycaemia: 63.3% vs. 41.5% (χ^2^ = 4.44, *p* = 0.035).

Symptom categories were not mutually exclusive. Jaundice was the most frequent presenting feature, occurring in 54 patients (47.4%), followed by abdominal pain in 50 (43.9%) and weight loss in 34 (29.8%); 15 patients (13.2%) had no tumour-related symptoms and were identified incidentally. Jaundice was significantly more common in the metastatic than the non-metastatic group (59.2% [29/49] vs. 38.5% [25/65]; χ^2^ = 4.02, *p* = 0.045; OR 2.32, 95% CI 1.09–4.95). In contrast, abdominal pain (53.1% [26/49] vs. 36.9% [24/65]; *p* = 0.13) and weight loss (24.5% [12/49] vs. 33.8% [22/65]; *p* = 0.38) did not differ significantly between groups; notably, weight loss was numerically more frequent in the non-metastatic group. Incidental detection was similarly distributed across stages (10.2% [5/49] vs. 15.4% [10/65]; *p* = 0.60). Symptom frequencies by stage are shown in Table 2 and Figure 1.

### 3.4. Admission Glucose and Disease Stage

Admission glucose analysed as a continuous variable did not differ significantly between the metastatic and non-metastatic groups (mean 148.4 ± 58.1 vs. 143.3 ± 61.5 mg/dL; median 136 vs. 123 mg/dL; Mann–Whitney U test, *p* = 0.29). By contrast, when the pre-specified threshold of ≥126 mg/dL was applied, hyperglycaemia was significantly more frequent in metastatic disease (63.3% [31/49], 95% CI 49.3–75.3, vs. 41.5% [27/65], 95% CI 30.4–53.7; χ^2^ = 4.44, *p* = 0.035; OR 2.42, 95% CI 1.13–5.19). Stage-specific hyperglycaemia frequencies are shown in Figure 1. The contrast between the null continuous comparison and the significant threshold-based comparison should be interpreted with caution: it is compatible with a threshold effect, but may also reflect the skewed distribution of glucose values and the loss of information inherent in dichotomisation [13].

### 3.5. Multivariable Analysis and Discriminative Performance

To determine whether the association between hyperglycaemia and metastatic stage was independent of demographic and metabolic confounders, multivariable logistic regression was performed (Table 3). After adjustment for age, sex, and diabetes status, admission hyperglycaemia ≥ 126 mg/dL remained independently associated with metastatic disease (aOR 2.59, 95% CI 1.13–5.95; *p* = 0.025), whereas age, sex, and diabetes were not. In a secondary model additionally including jaundice, neither hyperglycaemia (aOR 2.26, 95% CI 0.96–5.31; *p* = 0.061) nor jaundice (aOR 2.02, 95% CI 0.92–4.43; *p* = 0.079) retained statistical significance, consistent with partial overlap between the two variables and with limited statistical power. In a sensitivity model in which categorical hyperglycaemia was replaced by continuous glucose, no association with stage was observed (aOR 1.01 per 10 mg/dL, 95% CI 0.94–1.08; *p* = 0.80). The overall explanatory value of the models was low (pseudo-R^2^ 0.036 and 0.056, respectively).

**Figure 2:**
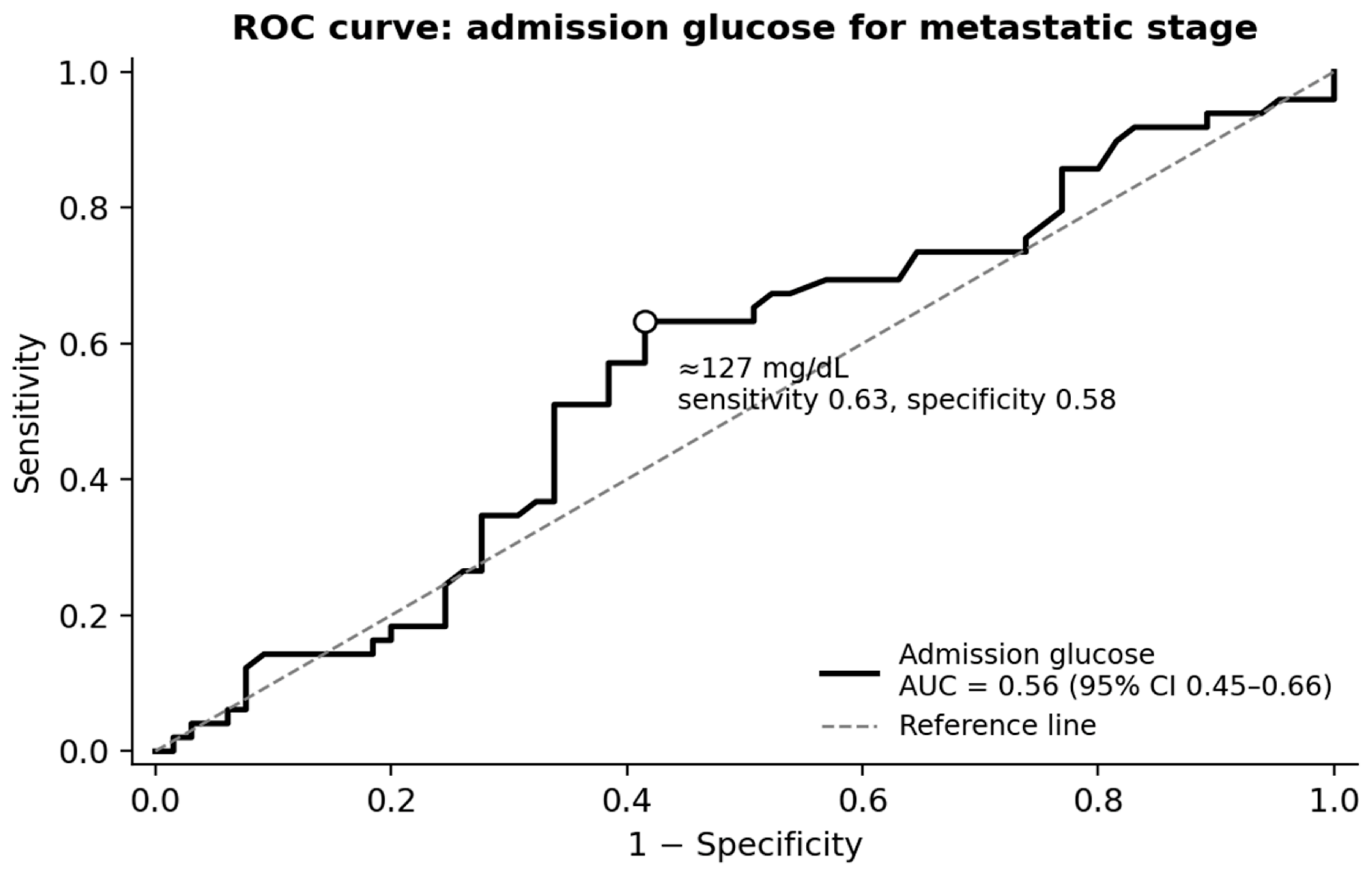
Receiver operating characteristic (ROC) curve for admission plasma glucose as a classifier of metastatic stage. The area under the curve was 0.56 (95% CI 0.45–0.66). The marked point indicates the cut-off identified by the Youden index (approximately 127 mg/dL; sensitivity 0.63, specificity 0.58). The diagonal reference line corresponds to no discrimination.

The discriminative performance of admission glucose for metastatic stage was assessed by ROC analysis (Figure 2). The area under the curve was 0.56 (95% CI 0.45–0.66); as the confidence interval includes 0.5, admission glucose alone did not discriminate reliably between metastatic and non-metastatic disease. The empirically optimal cut-off identified by the Youden index was approximately 127 mg/dL, closely matching the pre-specified threshold of 126 mg/dL, but its performance at this cut-off was modest (sensitivity 0.63, specificity 0.58, positive predictive value 0.53, negative predictive value 0.68). Admission glucose therefore shows a statistically detectable association with stage at the group level, but insufficient accuracy for classification of individual patients.

### 3.6. Co-Occurrence of Jaundice and Hyperglycaemia

**Figure 3:**
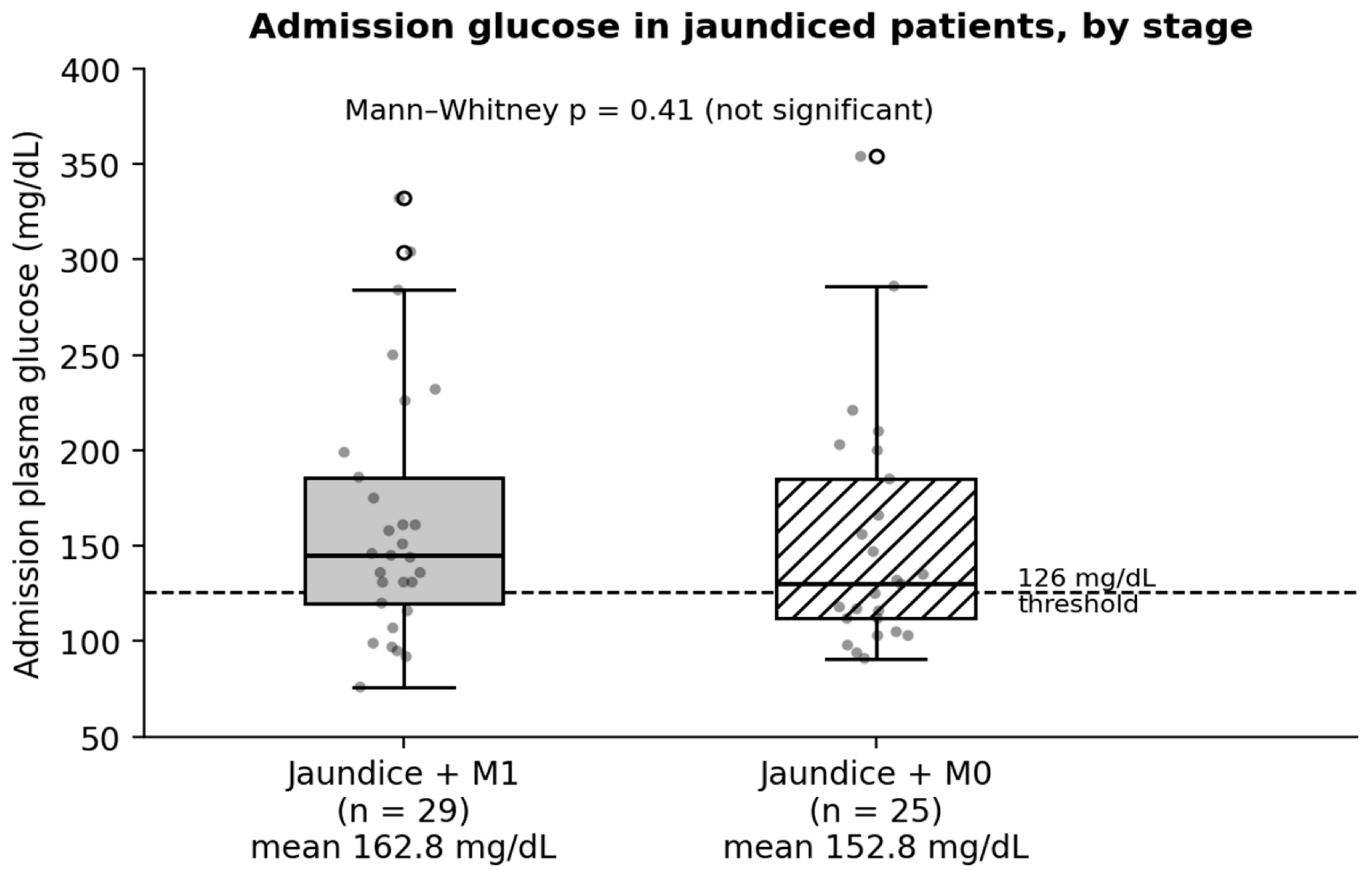
Admission plasma glucose in jaundiced patients, stratified by disease stage. Boxes show the median and interquartile range, whiskers the range within 1.5 × IQR, and points individual patients. The dashed line marks the 126 mg/dL hyperglycaemia threshold. The difference between groups was not statistically significant (Mann–Whitney U test, *p* = 0.41).

Among the 54 patients presenting with jaundice, mean admission glucose was 162.8 ± 65.4 mg/dL in those with metastatic disease (*n* = 29; median 145) and 152.8 ± 64.2 mg/dL in those with non-metastatic disease (*n* = 25; median 130). This difference of 10.0 mg/dL was not statistically significant (Mann–Whitney U test, *p* = 0.41), and the analysis was exploratory rather than pre-specified. Hyperglycaemia ≥ 126 mg/dL was present in 21 of 29 jaundiced patients with metastatic disease (72.4%) compared with 13 of 25 with non-metastatic disease (52.0%). These observations are descriptive; the subgroup sizes are small; no formal test of interaction between jaundice and hyperglycaemia was performed, and the co-occurrence pattern should not be interpreted as evidence of a combined marker effect (Figure 3).

## 4. Discussion

In this retrospective, single-centre cohort of 114 patients with newly diagnosed PDAC, jaundice at presentation and admission hyperglycaemia ≥ 126 mg/dL were each more frequent in patients with metastatic disease, and hyperglycaemia remained associated with metastatic stage after adjustment for age, sex, and diabetes. However, the magnitude of these associations was modest and the discriminative performance of admission glucose was poor (AUC 0.56, 95% CI 0.45–0.66). As an observational study, this analysis can establish association but not causation, and the findings should be regarded as hypothesis-generating rather than as evidence that these variables are clinically useful markers of stage.

### 4.1. Jaundice as Marker of Stage

Jaundice, resulting from biliary obstruction due to tumour infiltration or compression, occurred in nearly half of our patients and was significantly more frequent in metastatic disease (59.2% vs. 38.5%; *p* = 0.045). This is consistent with prior observations that jaundice may reflect tumour location in the pancreatic head with greater local extension or overall tumour burden [14]. The association was, however, of borderline statistical significance and did not persist in the multivariable model that also included hyperglycaemia, so it should be interpreted cautiously. It is also important to note the competing effect described previously: jaundice frequently prompts earlier presentation and can therefore accelerate diagnosis, which may partly offset its association with advanced disease [15]. Our data cannot separate these opposing influences.

### 4.2. Abdominal Pain and Weight Loss as Non-Discriminatory Symptoms

In contrast to jaundice, abdominal pain and weight loss showed no discriminatory value with respect to stage in our cohort, and weight loss was in fact numerically more frequent in the non-metastatic group. This apparent paradox merits comment. Both symptoms are highly non-specific and arise through mechanisms that are not stage-dependent: abdominal pain in PDAC results largely from perineural invasion, ductal obstruction, and local retroperitoneal infiltration, all of which can occur with small, localised tumours, while weight loss reflects exocrine insufficiency, malabsorption, anorexia, and cancer cachexia, which may be present early and progress independently of metastatic spread [4]. Both are also subject to substantial ascertainment bias in retrospective records, since their documentation depends on patient recall and clinician recording rather than on an objective measurement, whereas jaundice is a visible sign corroborated by biochemical cholestasis. The near-identical frequencies of abdominal pain and weight loss across stages therefore suggest that only symptoms with a direct anatomical correlate, such as jaundice, carry information about disease extent at presentation, and that symptom profile alone is an unreliable guide to stage.

### 4.3. Hyperglycaemia and Disturbed Glucose Metabolism

The relationship between pancreatic cancer and disturbed glucose metabolism is complex and bidirectional. Long-standing type 2 diabetes is an established risk factor for PDAC [16,17], whereas new-onset diabetes may represent an early paraneoplastic manifestation mediated by tumour-derived factors that promote insulin resistance and β-cell dysfunction, including adrenomedullin and the S100A8 N-terminal peptide [7,18], together with destruction of pancreatic parenchyma that impairs insulin secretion [8]. In our cohort, 40.4% of patients had coexisting diabetes and mean glucose was substantially higher in these patients, but the prevalence of diabetes did not differ between stage groups (42.9% vs. 38.5%; *p* = 0.78) and diabetes was not associated with metastatic stage in multivariable analysis. Notably, hyperglycaemia defined by the ≥126 mg/dL threshold was associated with metastatic disease while continuous glucose was not, which may indicate a threshold effect but may equally be an artefact of dichotomising a skewed variable [10]. Our findings are directionally consistent with studies reporting that glycaemic disturbance tracks with tumour burden [11], but the effect size we observed was smaller and, on ROC analysis, of no practical discriminative value. A key limitation in interpreting these data is that we could not distinguish new-onset from long-standing diabetes, since diabetes duration was documented in only nine of 46 patients and HbA1c in six of 114; this distinction is central to the paraneoplastic hypothesis and its absence prevents us from determining whether the hyperglycaemia we observed was tumour-induced or antecedent.

### 4.4. Co-Occurrence of Jaundice and Hyperglycaemia

Patients with jaundice and metastatic disease had numerically the highest mean admission glucose, but this difference was not statistically significant (162.8 vs. 152.8 mg/dL; *p* = 0.41). We therefore do not interpret this pattern as evidence of a synergistic or combined marker effect. Any of such co-occurrences could plausibly reflect a more advanced tumour process affecting both the biliary tree and glucose homeostasis, potentially compounded by hepatic metastases impairing hepatic glucose handling [19], but our data neither establish nor quantify this. Given the small subgroup sizes and the absence of a formal interaction test, the combination of jaundice and hyperglycaemia should be regarded as an observation requiring prospective evaluation rather than as a candidate stratification tool [11].

### 4.5. Clinical Implications

The implications of these findings are limited and must be framed cautiously. Our data do not support the routine use of admission glucose, alone or combined with symptom profile, to stratify individual patients by disease stage: the observed AUC of 0.56 indicates performance close to chance, and a positive predictive value of 0.53 at the 126 mg/dL threshold means means that roughly half of the patients classified as positive would, in fact, be misclassified. Practical recommendations for using this measurement in clinical decision-making would therefore be premature. What our findings do suggest is narrower. First, they add to evidence that glycaemic disturbance in PDAC is associated with tumour burden at the group level, supporting further mechanistic and prospective study of glucose metabolism as a correlate of disease extent. Second, because admission glucose and presenting symptoms are recorded universally and at no additional cost, they are pragmatic candidate variables for inclusion in future multivariable prediction models that combine clinical, laboratory, and imaging data, rather than as stand-alone markers [20]. Third, irrespective of any staging role, hyperglycaemia at admission is clinically relevant in its own right, as impaired glycaemic control may affect treatment tolerance, perioperative risk, and quality of life, and warrants diabetological assessment [21]. Any role for glucose measurement in guiding imaging or surveillance decisions remains unproven and would require prospective validation [22].

### 4.6. Limitations

Several limitations should be acknowledged. First, the retrospective design carries a risk of bias from incomplete or non-uniform documentation, particularly for subjective symptoms such as abdominal pain and weight loss, although strict inclusion criteria and independent dual review mitigated this. Second, the sample size was modest (*n* = 114, with 49 metastatic events) and was determined by the number of eligible cases at a single centre rather than by a formal power calculation; the study was therefore underpowered to detect small effects, to support reliable subgroup analyses, and to accommodate more than a small number of covariates in the regression models, and the wide confidence intervals reported throughout reflect this. Third, we used random admission glucose rather than fasting glucose or an oral glucose tolerance test, which introduces variability related to meal timing and metabolic state; although we excluded patients with sepsis, acute pancreatitis, ketoacidosis, and glucocorticoid therapy, we cannot exclude residual stress hyperglycaemia related to acute illness or hospitalisation. Fourth, HbA1c was available in only six of 114 patients and diabetes duration in only nine of 46 patients with diabetes, so we could not distinguish new-onset from long-standing diabetes, assess chronic glycaemic control, or evaluate the paraneoplastic hypothesis directly. Fifth, we could not systematically account for glucose-lowering therapy or for other medications and comorbidities influencing glycaemia, and no data were available on treatment received before diagnosis. Sixth, our analyses were univariate and multivariable but exploratory, without adjustment for multiple comparisons, and the multivariable models had low explanatory value (pseudo-R^2^ ≤ 0.06). Finally, the study was conducted at a single academic centre without external validation, which limits generalisability; the reported associations require confirmation in independent cohorts.

### 4.7. Future Directions

Future research should prospectively evaluate whether admission glycaemia and presenting symptoms contribute meaningfully to stage assessment in larger, multicentre cohorts with standardised glucose sampling. Such studies should record fasting glucose and HbA1c, document diabetes duration and treatment systematically to separate new-onset from long-standing diabetes, and pre-specify multivariable models with adequate events per variable. An integrated predictive model combining clinical, laboratory (including CA 19–9), and imaging parameters is more likely to achieve clinically useful discrimination than any single routine variable [13,23]. Mechanistic studies could further clarify the molecular basis of the PDAC–glucose relationship and identify candidate biomarkers [7]. Finally, whether early pancreatic imaging in patients with new-onset diabetes enables detection at a less advanced stage remains an important and unresolved question [22,24].

## 5. Conclusions

In this retrospective, single-centre cohort, jaundice at presentation and admission hyperglycaemia ≥ 126 mg/dL were each associated with metastatic stage in newly diagnosed PDAC, and hyperglycaemia remained associated after adjustment for age, sex, and diabetes (aOR 2.59, 95% CI 1.13–5.95). These associations were nevertheless modest, and admission glucose discriminated poorly between metastatic and non-metastatic disease (AUC 0.56, 95% CI 0.45–0.66). Abdominal pain and weight loss showed no association with stage. Accordingly, admission glucose and presenting symptoms cannot presently be recommended for stratifying individual patients by disease stage, and any such application would be premature. These findings are exploratory and hypothesis-generating; they establish association rather than causation and require confirmation in large, prospective, multicentre studies before any clinical role can be considered.

## Figures and Tables

**Table 1:** Baseline characteristics of the study cohort (*n* = 114).

Characteristic	Value
Women, *n* (%)	60 (52.6)
Men, *n* (%)	54 (47.4)
Median age, years (range)	68 (27–88)
Metastatic disease (M1), *n* (%)	49 (43.0)
Non-metastatic disease (M0), *n* (%)	65 (57.0)
Diabetes mellitus, *n* (%)	46 (40.4)
Type 2 diabetes, % of diabetes cases	87
Mean admission glucose, mg/dL (SD)	145.5 (59.8)
Median admission glucose, mg/dL	127

**Table 2:** Presenting symptoms overall and by disease stage.

Symptom	Overall, *n* (%)	Metastatic (M1), *n* = 49	Non-Metastatic (M0), *n* = 65	*p*-Value
Jaundice	54 (47.4)	29 (59.2)	25 (38.5)	0.045
Abdominal pain	50 (43.9)	26 (53.1)	24 (36.9)	0.13
Weight loss	34 (29.8)	12 (24.5)	22 (33.8)	0.38

Values are *n* (%). Percentages for the metastatic and non-metastatic columns are calculated within each stage group. Symptom categories are not mutually exclusive; patients could present with more than one symptom. *p*-values compare the metastatic (M1) and non-metastatic (M0) groups by the χ^2^ test with continuity (Yates) correction (jaundice χ^2^ = 4.02; abdominal pain χ^2^ = 2.34; weight loss χ^2^ = 0.76). Odds ratios (95% CI) for metastatic disease were 2.32 (1.09–4.95) for jaundice, 1.93 (0.91–4.10) for abdominal pain, and 0.63 (0.28–1.45) for weight loss.

**Table 3:** Multivariable logistic regression for metastatic disease (M1) as the dependent variable (*n* = 114; 49 events).

Variable	Adjusted OR	95% CI	*p*-Value
Hyperglycaemia ≥ 126 mg/dL	2.59	1.13–5.95	0.025
Age (per year)	1.00	0.97–1.04	0.89
Male sex	0.96	0.45–2.07	0.92
Diabetes mellitus	0.82	0.35–1.93	0.66

## Data Availability

The data presented in this study are available on reasonable request from the corresponding authors. The data are not publicly available owing to privacy and ethical restrictions.
